# Mushroom body defect is required in parallel to Netrin for midline axon guidance in *Drosophila*

**DOI:** 10.1242/dev.129684

**Published:** 2016-03-15

**Authors:** Marie-Sophie Cate, Sangeetha Gajendra, Samantha Alsbury, Thomas Raabe, Guy Tear, Kevin J. Mitchell

**Affiliations:** 1 MRC Centre for Developmental Neurobiology, New Hunts House, King's College, London, SE1 1UL, UK; 2 MSZ Universitat Würzburg, Versbacher Strasse 5, Würzberg 97078, Germany; 3 Howard Hughes Medical Institute, Division of Neurobiology, Department of Molecular and Cell Biology, University of California, Berkeley, Berkeley, CA 94720, USA; 4 Smurfit Institute of Genetics and Institute of Neuroscience, Trinity College Dublin, Dublin 2, Ireland

**Keywords:** *Drosophila*, Axon guidance, Midline, Mud, NuMA, LIN-5, Netrin

## Abstract

The outgrowth of many neurons within the central nervous system is initially directed towards or away from the cells lying at the midline. Recent genetic evidence suggests that a simple model of differential sensitivity to the conserved Netrin attractants and Slit repellents is insufficient to explain the guidance of all axons at the midline. In the *Drosophila* embryonic ventral nerve cord, many axons still cross the midline in the absence of the Netrin genes (*NetA* and *NetB*) or their receptor *frazzled*. Here we show that mutation of *mushroom body defect* (*mud*) dramatically enhances the phenotype of Netrin or *frazzled* mutants, resulting in many more axons failing to cross the midline, although mutations in *mud* alone have little effect. This suggests that *mud*, which encodes a microtubule-binding coiled-coil protein homologous to NuMA and LIN-5, is an essential component of a Netrin-independent pathway that acts in parallel to promote midline crossing. We demonstrate that this novel role of Mud in axon guidance is independent of its previously described role in neural precursor development. These studies identify a parallel pathway controlling midline guidance in *Drosophila* and highlight a novel role for Mud potentially acting downstream of Frizzled to aid axon guidance.

## Abstract (precis)

**Summary:** Mud/NuMA/LIN-5 is a signal transduction component, potentially acting downstream of Frizzled to mediate netrin-independent axon guidance towards the midline.

## INTRODUCTION

In the central nervous system (CNS) of vertebrates and invertebrates most neurons extend across the midline to form commissures, while the remainder extend on their own side (Tear, 1999; Kaprielian et al., 2001; Garbe and Bashaw, 2004; Evans and Bashaw, 2010). This decision depends in part on the responsiveness of the growth cone to Netrin attractants and Slit repellents, both secreted from cells at the midline, although additional mechanisms also exist to direct axons across the midline (Andrews et al., 2008; Dickson and Zou, 2010; Evans and Bashaw, 2010; Spitzweck et al., 2010; Organisti et al., 2015).

The Netrins act as chemoattractants to bring axons to the midline in flies, worms and vertebrates. In all these organisms, Netrin loss-of-function causes defects in the projection of axons towards the midline (Hedgecock et al., 1990; Harris et al., 1996; Mitchell et al., 1996; Serafini et al., 1996), and the same is true of mutations in the Netrin receptors *unc-40*, *DCC* and *frazzled* (Hedgecock et al., 1990; Kolodziej et al., 1996; Fazeli et al., 1997). However, their activity does not fully account for the guidance of all commissural axons across the midline, suggesting the existence of additional mechanisms (Brankatschk and Dickson, 2006).

In *Drosophila*, a number of components of Netrin-independent mechanisms that attract axons to the midline have been identified. Removal of either *Dscam*, *fmi* (*stan* – FlyBase) or *robo2* significantly enhances the failure of midline crossing caused by the absence of *frazzled* alone (Andrews et al., 2008; Spitzweck et al., 2010; Organisti et al., 2015). However, the loss of any of these genes individually does not lead to a significant midline guidance defect. Thus, the role of these additional pathways in directing commissural axons across the midline is only revealed in the absence of Netrin signalling.

Here we show that Mushroom body defect (Mud) also has a role in a Netrin-independent signalling pathway directing commissural axons to the midline in *Drosophila.* Mud has previously been identified to function within neuroblasts and sensory organ precursors to couple the orientation of the mitotic spindle to both intrinsic and extrinsic cues (Bowman et al., 2006; Izumi et al., 2006; Siller et al., 2006; Siller and Doe, 2009; Segalen et al., 2010). We show that its role in axon outgrowth is independent of its activity within neuroblasts. Mud is expressed within postmitotic neurons, where it may act downstream of Frizzled to influence intrinsic neuronal polarity necessary for axonal outgrowth.

## RESULTS AND DISCUSSION

### Netrin deficiencies reveal the presence of an additional activity mediating axon guidance across the midline

**Fig. 1.**
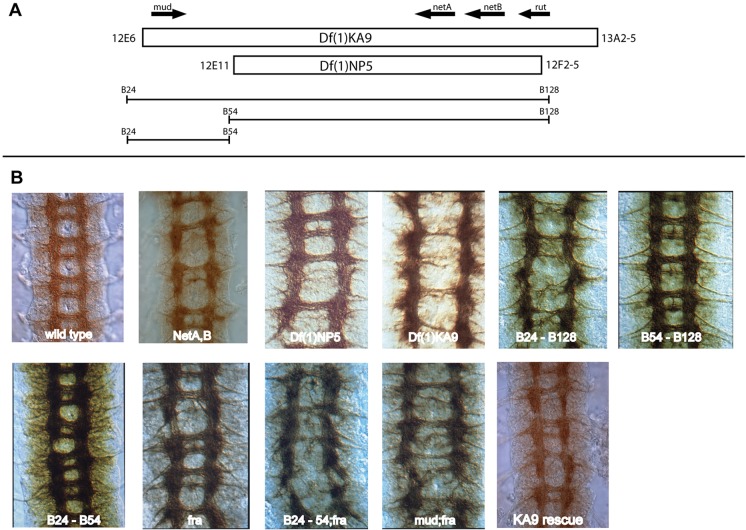
**Identification of Mud as an additional axon guidance factor required for commissure formation in the *Drosophila* CNS.** (A) Regions of the X chromosome deleted by the deficiencies *Df(1)KA9* and *Df(1)NP5* (boxes) used to remove the two Netrin genes *NetA* and *NetB.* Distal is to the left. Bracketed lines beneath represent the extents of the synthetic deficiencies used in this study that identify the location of an additional activity required for midline crossing distal to the Netrin genes. (B) *Drosophila* embryos immunostained with the CNS axon marker BP102. Anterior is up. In the wild-type embryo axon pathways extend in an orthogonal pattern with longitudinal tracts positioned either side of the midline and a pair of commissural tracts that connect the two sides of the nervous system within each segment. In embryos bearing double mutations for *NetA* and *NetB* commissure formation is disrupted, with fewer axons attracted across the midline, with the posterior commissure affected more severely. Embryos homozygous for a chromosomal deficiency, *Df(1)NP5*, that removes the Netrin genes have a phenotype similar to that of *NetA,B* animals, while the slightly larger deficiency *Df(1)KA9* has a stronger BP102 phenotype with fewer axons attracted to the midline, suggesting that an additional activity has been removed. The synthetic deficiency *Df(1)B24-B128* that is deficient for the Netrin genes and a distal region displays the stronger phenotype. Embryos deficient for the distal region alone, *Df(1)B24-B54*, display very little disruption to the axon pathways. Mutations that remove the attractive Netrin receptor *frazzled* (*fra*) have a similar phenotype to that of embryos lacking the Netrin genes. When loss of *fra* is combined with removal of the distal material in *Df(1)B24-54;fra*, this causes the increased midline crossing failure phenotype. *mud* is a candidate gene for the additional activity removed in *Df(1)B24-54*, and *mud;fra* double mutants display the same enhanced phenotype as *Df(1)KA9* embryos. Reintroduction of *mud* as a transgene into *Df(1)KA9* embryos reverts the midline phenotype to that seen when the Netrin genes are removed alone and also rescues the mild phenotype seen in *mud* mutant animals, confirming that *mud* encodes the additional midline attractive activity.

*Drosophila* has two Netrin genes, *NetA* and *NetB*, which are adjacent on the X chromosome (Fig. 1A). The original studies investigating the role of the *Drosophila* Netrins reported a difference in phenotype between a small deficiency *Df(1)NP5* that removed both Netrin genes and a slightly larger deficiency, *Df(1)KA9*, that extends further than NP5 (Fig. 1A) (Harris et al., 1996; Mitchell et al., 1996). Embryos hemizygous for the smaller deficiency *Df(1)NP5* have thinner or occasionally absent axon commissures in the ventral nerve cord, with the posterior commissure being more strongly affected, and occasional breaks in the longitudinal connectives (Fig. 1B, Table 1). This phenotype is similar to that seen in embryos where only *NetA* and *NetB* have been removed (Fig. 1B, Table 1) (Brankatschk and Dickson, 2006), although *Df(1)NP5* is slightly more severe (Andrews et al., 2008). By contrast, embryos hemizygous for the slightly larger deficiency *Df(1)KA9* exhibit a more severe phenotype, with a near complete loss of midline crossing in some commissures (Fig. 1B, Table 1). The larger deficiency affects the guidance of anterior and posterior commissural axons at the midline.

**Table 1.**
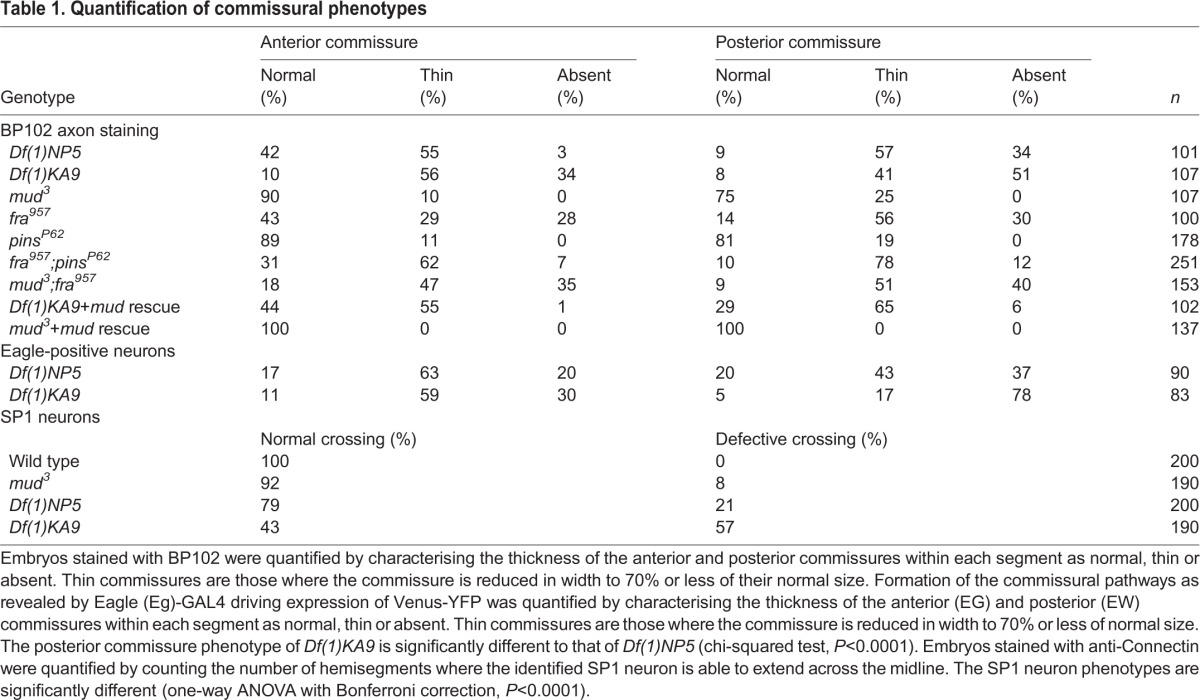
**Quantification of commissural phenotypes**

Restoration of either Netrin gene at the midline is sufficient to completely rescue the *Df(1)NP5* phenotype, while rescuing the *Df(1)KA9* to near wild type (Harris et al., 1996; Mitchell et al., 1996). These findings indicate the existence of a gene activity, also deleted in *Df(1)KA9*, that enhances midline crossing defects caused by the absence of the Netrins, but which has a mild phenotype when removed alone.

**Fig. 2.**
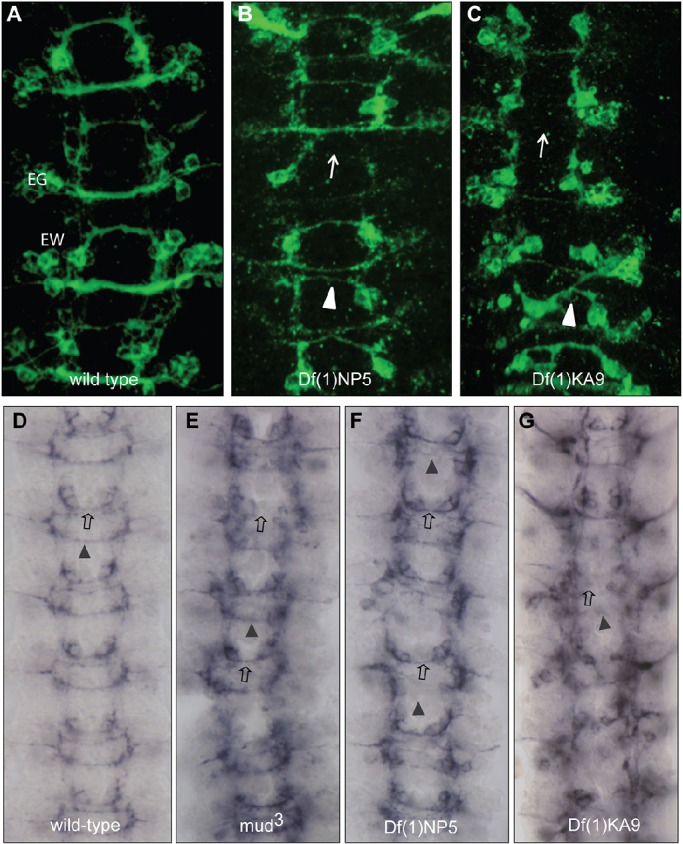
**Loss of *mud* enhances axon outgrowth defects at the CNS midline in Netrin-deficient embryos.** (A) Eagle-positive axons extend in the anterior (EG) and posterior (EW) commissures at the midline of the CNS. (B) Upon loss of Netrin signalling [*Df(1)NP5*] there is a reduction in the ability of Eagle-positive axons to cross the midline, leading to a thinning of commissures (arrowhead) or complete loss of midline crossing (arrow). (C) Loss of both Mud and Netrin activity [*Df(1)KA9*] results in a greater disruption of midline crossing, with many axons failing to cross in both anterior and posterior commissures (arrow) or taking aberrant trajectories (arrowhead). (D) Anti-Connectin reveals SP1 axons that extend in the anterior commissure (open arrow) and additional axons extending in the posterior commissure (arrowhead). (E) Loss of *mud* alone leads to mild defects, with 7% of segments showing failure of SP1 axons to cross. (F) Loss of Netrin signalling results in increased disruption, with axons failing to cross in both anterior (open arrow) and posterior (arrowhead) commissures. (G) Removal of both Mud and Netrin signalling leads to an enhanced phenotype in which midline crossing in the posterior commissure is severely affected and there is an increase in the failure of SP1 axons to cross the midline.

Markers for specific subsets of commissural neurons confirm the increased severity of commissural defects in *Df(1)KA9* compared with *Df(1)NP5* embryos (Table 1, Fig. 2). The Eg-GAL4 driver identifies the EG cluster of 10-12 cells that extend axons in the anterior commissure and the EW cluster of four cells that project in the posterior commissure (Higashijima et al., 1996; Dittrich et al., 1997; Garbe and Bashaw, 2007). Midline crossing by Eagle-positive EG and EW neurons is significantly more disrupted in *Df(1)KA9* than in *Df(1)NP5*, due both to stalling of axons prior to midline crossing and to misguidance, where axons extend across the midline along an aberrant trajectory (Table 1, Fig. 2). In *Df(1)NP5* embryos the EW axons fail to cross the midline in 37% of segments, while 20% of the EG axons do not cross. In *Df(1)KA9* embryos the number of segments where EW axons fail to cross the midline is increased to 78% (Fig. 2). The outgrowth of the SP1 neuron, one of the earliest axons to cross the midline in the anterior commissure, was examined using anti-Connectin (Meadows et al., 1994). Behaviour of the SP1 neurons mirrors that of the EG axons, with significantly more failing to cross the midline in *Df(1)KA9* (57%) compared with *Df(1)NP5* (21%).

### Mud is the enhancer of Netrins

We used overlapping synthetic deficiencies in the region (Livingstone, 1985) to map the enhancer activity (Fig. 1A). *Df(1)B24-B128*, which deletes both *NetA* and *NetB* plus distal material, displays the stronger axon guidance phenotype suggesting that the gene responsible lies distal to *NetA.* The synthetic deficiency *Df(1)B24-B54* selectively removes this distal genetic material – which includes a candidate gene, *mushroom body defect* (*mud*), and a small number of additional genes – while leaving the Netrin genes intact. Embryos hemizygous for this deficiency display a subtle CNS axon pathway phenotype. There is a general but weak irregularity in the usually orthogonal organisation of axon tracts as revealed by BP102 staining, with occasionally thinner commissures and rare breaks in the longitudinals. This phenotype is indistinguishable from that observed in embryos hemizygous for any of several alleles of *mud* (Fig. 1B).

To test whether *mud* encodes the additional midline guidance activity removed in *Df(1)KA9* we examined embryos double mutant for *mud* and *frazzled* (*fra*). *fra* embryos have a similar commissural axon guidance phenotype to small Netrin deficiencies (Fig. 1B) (Kolodziej et al., 1996). When *mud* alleles, or the small deficiency *Df(1)B24-B54* that deletes the *mud* region, are combined with *fra* alleles the double-mutant embryos fail to form the majority of commissures – a phenotype indistinguishable from that of *Df(1)KA9*.

Confirmation that *mud* is necessary for the formation of the commissures that form in *Df(1)NP5* embryos was demonstrated by reintroducing *mud* as a transgene into *Df(1)KA9* embryos, using a BAC construct that contains the *mud* genomic region. This resulted in a rescue of the BP102 phenotype in *Df(1)KA9* embryos to one resembling that of the smaller *Df(1)NP5* deficiency (Fig. 1B). Thus, *mud* encodes the enhancer activity that accounts for the more severe phenotype observed in the larger *Df(1)KA9* deficiency and is necessary to enable axons to cross the midline in this background. This places Mud as a component within an additional signalling pathway that directs axons to the midline, the role of which becomes apparent in the absence of Netrin signalling.

### *mud* mutation has direct effects on axon extension and guidance

**Fig. 3.**
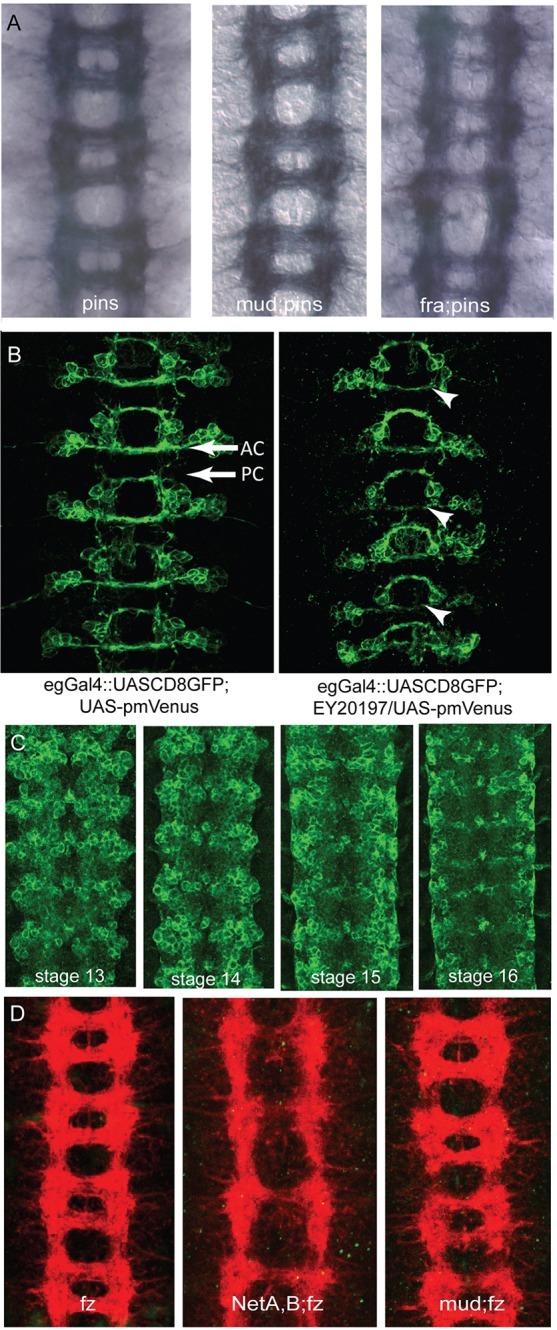
**Mud acts in neurons and its role in axon guidance is independent of Pins.** (A) Mud has previously been identified to function in a Partner of inscuteable (Pins)-dependent pathway within neuroblasts. In common with the loss of *mud*, absence of *pins* does not lead to significant axon guidance deficits as revealed by the BP102 antibody. Embryos deficient for *mud* and *pins* also show no outgrowth defects. Absence of *pins* does not enhance the axon guidance defects associated with loss of *fra*, suggesting that Mud acts in a Pins-independent pathway to enhance the axon guidance defects caused by a loss of Netrin signalling. (B) Mud overexpression in Eagle-positive neurons causes a reduction of midline crossing by the EG neurons (arrowheads), which cross through the anterior commissure (AC), revealing that Mud can influence the guidance of neurons. PC, posterior commissure. (C) Venus-YFP-tagged Mud protein driven by the *mud* promoter is expressed widely within the CNS at stage 13 and becomes restricted to subsets of neurons and glia by stage 16. (D) Mud has been found to act downstream from Frizzled (Fz). Loss of *fz* has little impact on axon outgrowth at the midline as revealed by BP102, yet the double mutant *NetA,B;fz* is as severe as *Df(1)KA9* or *mud;fra.* This suggests that Mud and Fz might act in the same pathway, which is supported by the fact that a *mud;fz* mutant phenotype resembles that of *mud* or *fz* single mutants.

Mud has previously been shown to be required during the asymmetric division of embryonic neuroblasts, where it couples mitotic spindle orientation to cortical polarity at metaphase (Bowman et al., 2006; Izumi et al., 2006; Siller et al., 2006). Initial defects in this process in *mud* mutants are largely recovered by a realignment of the spindle during telophase, and only minor consequences have been reported on subsequent neuronal number and fate. The pattern of expression of Even-skipped is largely unchanged in *mud* mutants, *Df(1)KA9* or *Df(1)NP5* (Izumi et al., 2006) (data not shown). Similarly, the neurons identified by anti-Futsch (22C10) and anti-Fasciclin 2 (1D4) form as normal (data not shown), indicating little or no change in cell fate. To test further whether the roles of Mud during neuroblast division and axon outgrowth are separable, the ventral nerve cord phenotypes of *fra;pins* double mutants were examined. Pins functions with Mud in asymmetric neuroblast division (Siller et al., 2006) and if loss of *mud* during neuroblast division leads to defects in subsequent axon outgrowth, then a *pins* mutant should have a similar effect on axon guidance and would enhance *fra* phenotypes*.* However, the ventral nerve cord phenotype of *fra;pins* double mutants is no more severe than that of *fra* mutants alone (Fig. 3A). The effects on axon guidance due to the absence of Mud are thus not attributable to a disruption of cell fate.

We also examined the consequence of manipulating the levels of Mud activity within neuroblasts and neurons using the UAS-GAL4 system (Brand and Perrimon, 1993). We made use of a P-element insertion (EY20197) that inserts a UAS immediately upstream of *mud.* Increasing Mud expression in neuroblasts using the Sca-GAL4 driver did not result in axon outgrowth defects (data not shown). However, increasing Mud expression in Eagle-positive neurons caused a reduction in the number of axons extending across the midline through the anterior commissure (Fig. 3B) suggesting that Mud acts in a dose-dependent manner in axons. The decrease in axon number is not due to a loss of the cells. This disruption is consistent with a direct role of Mud in axonal projection or guidance in postmitotic neurons.

### Mud is expressed in postmitotic neurons

Mud contains multiple coiled-coil domains and a microtubule-binding domain and shares similarity to the vertebrate protein NuMA (Numa1) and LIN-5 of *C. elegans* (Guan et al., 2000; Bowman et al., 2006; Izumi et al., 2006; Siller et al., 2006). The *Drosophila* gene encodes seven isoforms, and a probe that detects all isoforms shows that mud transcripts are expressed throughout embryonic development. Zygotic expression is restricted to the ventral nerve cord from stage 11 and *mud* remains expressed in the ventral nerve cord until the end of embryogenesis.

Mud protein expression has previously been described as localised to both the apical cortex and the centrosome of neuroblasts (Izumi et al., 2006). We find that Mud is also expressed within neurons, where it is localised within a punctate pattern within the soma. Mud is expressed in most, if not all, neurons and is also present within midline cells and members of the longitudinal glia (Fig. 3C). NuMA has similarly been reported to be expressed in a particulate distribution within the somatodendritic compartment of postmitotic sympathetic and hippocampal neurons, a distribution that requires intact microtubules (Ferhat et al., 1998). Mud, in common with its LIN-5 and NuMA homologues, is able to bind microtubules, has a conserved role in regulating mitotic spindle formation and is able to link intrinsic or extrinsic cues to orient spindle formation (Du et al., 2001; Segalen et al., 2010). Mud also has an ability to recruit dynein/dynactin (Siller and Doe, 2009) and functions in the planar cell polarity pathway (Segalen et al., 2010; Johnston et al., 2013), raising the possibility that Mud might function in neurons to link polarity information with the dynein/dynactin complex to orient microtubule structures within neurons to encourage directed outgrowth.

### Commissure formation utilises a variety of partially redundant pathways

Multiple signalling pathways, in addition to Netrins, cooperate to direct axon outgrowth towards and across the midline. Mutations in the genes encoding the transmembrane proteins Dscam1, Robo2 and Fmi or the intracellular proteins Abl and Trio and now Mud can all dramatically enhance the reduction of axonal midline crossing in Netrin or *fra* mutants (Forsthoefel et al., 2005; Andrews et al., 2008; Spitzweck et al., 2010; Evans et al., 2015; Organisti et al., 2015).

Because Mud has previously been implicated in a signalling pathway downstream of Frizzled (Fz) in planar cell polarity (Segalen et al., 2010), we tested for genetic interactions between *fz* and Netrins and between *fz* and *mud*. We find, as recently independently reported (Organisti et al., 2015), that *NetA,B;fz* double mutants display a severe lack of commissures, similar to *mud;fra* or *Df(1)KA9* mutants, whereas *mud;fz* mutants are not appreciably more severe than either *mud* or *fz* mutants alone (Fig. 3D). These data are consistent with a model whereby Fz and Mud both operate in a common, parallel pathway to the Netrins, possibly in concert with Fmi (Organisti et al., 2015). Double mutants of *mud* with *Robo2* or with *Dscam1* did not show a significant increase in midline crossing defects (data not shown), which formally suggests that Mud might also act in common pathways with these proteins, but this straightforward interpretation is complicated by the multiple functions demonstrated for Robo2 and Dscam1 in midline guidance (Andrews et al., 2008; Evans et al., 2015).

Further investigations are necessary to gain a better understanding of the mechanisms through which these multiple pathways are integrated within growth cones to enable the precise navigation of commissural axons at the midline.

## MATERIALS AND METHODS

### Genetics

The following *Drosophila* stocks were used: (1) *mud^1^/Fm7cβGal*, (2) *mud^3^/Fm7cβGal*, (3) *fra^957^/CyWglacZ*, (4) *Df-NP5/Fm7cβGal*, (5) *Df-KA9/Fm7cβGal*, (6) *NetA,B/Fm7βactin* (courtesy of B. Dickson, IMP, Vienna), (7) *fz^1^/Tm6bAbdAlacZ*, (8) *mud^3^/Fm7c;fra^957^/CyOwgβGal*, (9) *Pins^P62^/Tm6bAbdalacZ*, (10) *fra^957^/CyOwgβGal;PinsP62/Tm6bAbdalacZ*, (11) *mud^3^/Fm7cβGal;Pins^P62^/Tm6bAbdalacZ*, (12) *P{EPgy2}EY20197*, (13) *egGal4::UASCD8GFP*, (14) *elavGal4* on II, (15) *egGal4*, (16) *Sca-GAL4*, (17) *mud^3^/Fm7kr::GFP* and (18) *fra^957^/CyODfdEYFP*. X;Y translocation stocks with breakpoints in the 12E-13A region, originally constructed by Stewart and Merriam (1973), were used to generate deficiencies for defined regions of the X chromosome as described by Ashburner (1989). Unless otherwise stated, stocks were obtained from the Bloomington Stock Center.

### Molecular biology

A genomic rescue construct for *mud* was created in P[acman] (Venken et al., 2006). 17.5 kb was retrieved from BAC CH322-147E14 (BACPAC Resources Center) (Venken et al., 2009) covering chromosome arm X from 14138384 to 14157868. The rescue construct includes the promoter region of *mud*, located 147 bp downstream of CG32599 to 1546 bp upstream of *mud*, the *mud* gene and region downstream to 363 bp upstream of the closest downstream gene, CG1461. 500 bp homology arms homologous to the left and right ends of the transgene were subcloned into P[acman] to create a targeting construct. MW005 cells [courtesy of Colin Dolphin, King's College, London; described in Westenberg et al. (2010)] were made competent for recombineering by inducing the Red recombinase essentially as described (Dolphin and Hope, 2006). After verification of the correct integration of genomic DNA into P[acman] by sequencing, transgenic flies containing this P[acman]-mud construct inserted into the VK6 attP site at 19E7 on the X chromosome were obtained by BestGene. A Venus-YFP tag was inserted at the N-terminus of Mud using Gibson assembly and the construct inserted at attP40 by BestGene.

### Immunochemistry

Embryos were collected, fixed and stained as previously described by Patel (1994). The following primary antibodies were used: monoclonal antibody BP102 (Developmental Studies Hybridoma Bank; 1:20), mouse anti-β-Gal (Promega, Z3781; 1:300), mouse anti-Connectin [courtesy of Robert White, University of Cambridge, UK (Meadows et al., 1994); 1:20] and rabbit anti-GFP (ThermoFisher Scientific, A6455; 1:300). Secondary antibodies were purchased from Molecular Probes. Stacks of images were obtained using a Zeiss LSM 510 confocal microscope and processed using Volocity 5.2 imaging software (Improvision).
